# Editorial: Multiscale anatomy and biophysics of the autonomic nervous system: implications for neuromodulation

**DOI:** 10.3389/fnins.2023.1289177

**Published:** 2023-11-01

**Authors:** Nicole A. Pelot, Marmar Vaseghi, Leah Reznikov, Peregrine B. Osborne, Silvia V. Conde

**Affiliations:** ^1^Department of Biomedical Engineering, Duke University, Durham, NC, United States; ^2^UCLA Cardiac Arrhythmia Center, Division of Cardiology, Department of Medicine, University of California, Los Angeles, Los Angeles, CA, United States; ^3^Department of Physiological Sciences, University of Florida, Gainesville, FL, United States; ^4^Department of Anatomy and Physiology, University of Melbourne, Melbourne, VIC, Australia; ^5^NOVA Medical School, Faculdade de Ciências Médicas, Universidade NOVA de Lisboa, iNOVA4Health, Lisboa, Portugal

**Keywords:** autonomic nervous system, neuroanatomy, neuromodulation, neural stimulation, imaging, image analysis, cardiovascular disease

## Introduction

Using peripheral neuromodulation to target the autonomic nervous system is a rapidly growing therapeutic approach in which electrical, ultrasonic, or optical signals are delivered invasively or non-invasively. These signals alter nerve activity to treat multiple visceral system disorders, including cardiovascular, respiratory, gastrointestinal, and urogenital pathologies. Given that the neuromodulation targets include multiple fiber types that innervate multiple organ systems, there is a critical need to better understand the relevant neuroanatomy and physiology of this network to develop more effective clinical therapies.

This Research Topic introduces studies that advance our knowledge of neuroanatomy, including functional organization of the pig vagus nerve, innervation of the kidneys in mice, and a review of cardiac vagal afferents. Additional studies provide analytical tools to quantify the structure of autonomic nerves, including efficient segmentation of microCT of the human vagus nerve and statistical descriptions of the spatial arrangement of axons. Finally, neuromodulatory approaches for treatment of ischemia are presented.

## Functional organization of the pig vagus nerve

Implanted vagus nerve stimulation (VNS) is most commonly used to treat epilepsy and is under investigation for many other conditions. Thompson et al. used complementary approaches to reveal the functional organization of the left cervical vagus nerve (VN) in anesthetized pigs: electrical impedance tomography (EIT), electrical stimulation using small contact pairs, and microCT of post-mortem nerve samples. Microcomputed tomography (microCT) uses X-rays for 3D, non-destructive, high resolution (e.g., 150 μm voxels) imaging of stained tissues. Based on the microCT, at the mid-cervical level of the VN, fibers from pulmonary and recurrent laryngeal (RL) branches were mixed across many fascicles (i.e., bundles of axons), although they spanned mostly separate regions, while the cardiac fascicles remained distinct; the rotational location of the three functional groupings was approximately consistent across animals. In the EIT study, they delivered current through different contact pairs of a multi-contact cuff electrode; the voltage recorded on all contacts was used in image reconstruction to estimate the locations of neural activity in the nerve cross section. Correlating the locations of cardiac, pulmonary, and RL fascicles from microCT with changes in heart rate, respiratory rate, and laryngeal EMG, respectively, demonstrated that EIT and selective stimulation could approximately localize these functional groupings. Key remaining knowledge gaps include analogous data for the right VN, the organization of vagal fibers at the cervical level that innervate abdominal organs, and functional organization of the human VN, for which the fascicles are fewer, larger, and split/merge more frequently (Pelot et al., 2020; Upadhye et al., 2022).

## Afferent innervation of glomeruli in mouse kidneys

Recent advances in treatment of hypertension include ablation of nerves innervating the kidneys; however, anatomy and function of afferent fibers in renal nerves are largely unknown. Tyshynsky et al. analyzed the anatomical relationship between afferent nerve fibers and renal glomeruli in mice using higher-throughput imaging for population counts and higher-resolution imaging for distance measurements. Renal glomeruli are specialized blood vessels that filter blood in the kidneys to create urine. The afferents were visualized using immunolabeling of calcitonin gene-related peptide (CGRP+) in samples from wildtype and transgenic mice, as well as fluorescence in TRPV1 lineage cells (tdTomato+) of transgenic mice. Approximately half of glomeruli had nearby CGRP+ or tdTomato+ axons, with higher likelihood for glomeruli deeper in the cortex. Bowman's capsule surrounds the glomerular capillary loops to filter blood and provide passage toward the ureters and urinary bladder. The afferents seemed to travel along the surface of Bowman's capsule, and thus they may transmit changes in glomerular pressure for reflexive responses to adjust vascular diameter. Additional studies are needed to confirm direct innervation and to elucidate the transduction and signaling roles of afferents in renal (patho)physiology.

## Review of cardiac vagal afferents

Visceral organ injury and disease can cause pathological changes in the autonomic nervous system, which can in turn, affect visceral organ function. These pathological changes present an important consideration when evaluating therapeutic strategies using bioelectronic medicine to target the autonomic system, as highlighted by Van Weperen and Vaseghi. In this review, the authors summarize the role of cardiac vagal afferent/sensory neurotransmission in health, and they highlight important reported alterations in signaling during cardiovascular disease. They further demonstrate how a detailed mechanistic understanding of cardiac physiology and pathophysiology, combined with a wide range of advanced, clinically relevant animal models and techniques (e.g., Veerakumar et al., 2022) can provide unique model systems for investigating how to increase the therapeutic efficacy of targeted autonomic neuromodulation strategies under clinically-relevant conditions.

## Efficient segmentation of fascicles from microCT of human vagus nerves

MicroCT imaging plays a pivotal role in unraveling the intricate three-dimensional arrangement of the fascicles of the human vagus nerve, a critical element for anatomical exploration and the progress of neuromodulation therapies. However, conventional manual segmentation methods to identify the fascicle boundaries can be laborious. In this issue, Buyukcelik et al. developed a U-Net convolutional neural network to automate the segmentation of fascicles within microCT images of the human vagus nerve. The U-Net structure was originally designed for segmentation of biomedical images (Ronneberger et al., 2015). With this U-Net, the authors accurately segmented approximately 500 images of a cervical vagus nerve in just 24 s. This study enables the use of deep learning fascicle segmentation of microCT images by employing a conventional U-Net as a benchmark. Although it can be further optimized, the U-Net described by Buyukcelik et al. will contribute to generating data to enrich computational models, thereby facilitating the advancement of analysis and the strategic design of neuromodulation therapies tailored specifically for the human vagus nerve.

## Spatial arrangement of axons in autonomic nerves

Shemonti et al. report statistical methods for quantifying the spatial arrangements of axons in peripheral nerves, accounting for their intensity, interaction, and regionality. They used a published dataset of rat pelvic and vagus nerves imaged with transmission electron microscopy from which unmyelinated axons were delineated through manual segmentation. They present methods for spatial point pattern construction (an analysis used in many fields to measure spatial distribution and locations in two- or three-dimensional space), feature configuration (a set of features), and Sinkhorn distance computation (a mathematical/statistical tool for optimizing transport/distance between objects), encompassed in a generalized pipeline to analyze the inhomogeneous structure of peripheral nerves. Though the differences noted between the vagus and pelvic nerves and between nerve samples from male and female rats are of unknown physiological and biological significance, their method provides an objective technique applicable to quantifications of axonal spatial arrangements in any nerve.

## Stimulation of the dorsal root ganglion to treat cardiac sympathetic hyperactivity

Sustained sympathoexcitation can cause ventricular arrhythmias and sudden cardiac death. Kuwabara et al. examined neuromodulation of the dorsal root ganglion (DRG) as a means to dampen cardiac sympathoexcitation and ventricular excitability. Using a porcine model of acute myocardial ischemia (i.e., obstructed blood flow to the heart muscle), they found that DRG stimulation reduced sympathoexcitation caused by cardiac ischemia, consistent with their observation of reduced markers of neuronal activation in the dorsal horn of the spinal cord. Combined, these findings lay the preclinical groundwork to suggest that DRG stimulation may have important cardioprotective effects in humans, though additional data are required to know the extent and degree of potential benefit.

## Stimulation of the spinal cord to treat cardiac sympathetic hyperactivity

Using a similar model of porcine myocardial ischemia, Salavatian et al. examined the potential of preemptive spinal cord stimulation to reduce sympathoexcitation and ventricular excitability caused by myocardial ischemia. The authors found that preemptive stimulation of the dorsal horn of the spinal cord reduced activity of ischemia-sensitive neurons during myocardial ischemia. Preemptive stimulation also reduced the firing activity of neurons within the intermediolateral column of the spinal cord, and it prevented the myocardial ischemia-induced augmentation of synchrony between dorsal horn and intermediolateral column neurons—where the intermediolateral column contains the cell bodies of neurons of the sympathetic nervous system. Though Salavatian et al. observed benefits in their study, additional studies are necessary to better understand the potential of spinal cord stimulation as a therapeutic intervention for cardiac diseases in humans and animals.

## Conclusions

The studies highlighted in this special issue span two overlapping themes: (1) quantification of the anatomy of the peripheral autonomic nervous system at different scales, and (2) cardiovascular anatomy and neural stimulation treatments. The anatomical studies spanned functional organization of the pig vagus nerve, proximity of afferent fibers to glomeruli in mouse kidney, a review of cardiac vagal afferents, segmentation of fascicles in human cervical vagus nerve, and statistical descriptors for arrangement of axons in peripheral nerves. The latter two tools for image analyses can be applied to other parts of the peripheral nervous system. The cardiovascular studies spanned the role of kidneys in hypertension, locations of cardiac fibers in the mid-cervical pig vagus nerve, a review of cardiac vagal afferents in health and disease, as well as stimulation of either the dorsal root ganglion or spinal cord to treat cardiac sympathetic hyperactivity. While these studies advance our understanding of the autonomic nervous system, significant knowledge gaps remain in order to selectively apply neuromodulatory therapies at targeted nexus points to treat specific organ pathologies while reducing off-target effects.

## Author contributions

NP: Conceptualization, Writing—original draft, Writing—review and editing. MV: Writing—original draft, Writing—review and editing. LR: Writing—original draft, Writing—review and editing. PO: Writing—original draft, Writing—review and editing. SC: Writing—original draft, Writing—review and editing.
